# Comparison of tooth movement and biological response resulting from different force magnitudes combined with osteoperforation in rabbits

**DOI:** 10.1590/1678-7757-2020-0734

**Published:** 2021-03-25

**Authors:** Cheng-Yi HUANG, Hai-Ping LU, Yu-Feng YU, Xi DING, Zan-Zan ZHANG, Jia-Nan ZHANG

**Affiliations:** 1 Zhejiang University School of Medicine Sir Run Run Shaw Hospital Hangzhou China Zhejiang University School of Medicine, Sir Run Run Shaw Hospital, Department of Dentistry, Center of Orthodontics, Hangzhou, China.; 2 Zhejiang Chinese Medical University College of Stomatology Department of Orthodontics Hangzhou China Zhejiang Chinese Medical University, College of Stomatology, Department of Orthodontics, Hangzhou, China.; 3 Hangzhou Cancer Hospital Department of Radiotherapy Hangzhou China Hangzhou Cancer Hospital, Department of Radiotherapy, Hangzhou, China.; 4 The First Affiliated Hospital Wenzhou Medical University Department of Stomatology Wenzhou China The First Affiliated Hospital of Wenzhou Medical University, Department of Stomatology, Wenzhou, China.

**Keywords:** Osteoperforation, Tooth movement, Biological response, Force

## Abstract

**Objective:**

To compare tooth movement rate and histological responses with three different force magnitude designs under osteoperforation in rabbit models.

**Methodology:**

48 rabbits were divided into three groups: Group A, Group B, and Group C, with traction force of 50 g, 100 g, 150 g, respectively. Osteoperforation was performed at the mesial of the right mandibular first premolar, the left side was not affected. One mini-screw was inserted into bones between two central incisors. Coil springs were fixed to the first premolars and the mini-screw. Tooth movement distance was calculated, and immunohistochemical staining of PCNA, OCN, VEGF, and TGF-β1 was analyzed.

**Results:**

The tooth movement distance on the surgical side was larger than the control side in all groups (P<0.01). No significant intergroup difference was observed for the surgical side in tooth movement distance among the three groups (P>0.05). For the control side, tooth movement distance in Group A was significantly smaller than Groups B and C (P<0.001); no significant difference in tooth movement distance between Group B and Group C was observed (P>0.05). On the tension area of the moving premolar, labeling of PCNA, OCN, VEGF and TGF-β1 were confirmed in alveolar bone and periodontal ligament in all groups. PCNA, OCN, VEGF and TGF-β1 on the surgical side was larger than the control side in all groups (P<0.001).

**Conclusion:**

Osteoperforation could accelerate orthodontic tooth movement rate in rabbits. Fast osteoperforation-assisted tooth movement in rabbits was achieve with light 50 g traction.

## Introduction

In clinical tooth extraction cases of protrusive adult orthodontics, it always takes approximately two and half years of traditional fixed appliance treatment. Long-term comprehensive orthodontic treatments bring many risks to patients, such as root absorption, dental decalcification and periodontal disease.^1^ Thus, the efforts to increase the rate of tooth movement and shorten the treatment period have always been a focus of clinical orthodontics. Furthermore, the growing number of adults requiring orthodontic treatment has brought more awareness to the matter.^2^

In the last 20 years, many procedures to accelerate tooth movement and shorten the orthodontic treatment period, such as low-intensity laser treatments, low-intensity pulsed ultrasound, pulsed electromagnetic fields, corticotomy, and corticision, have been investigated.^3,4^ Good-quality randomized clinical trials show that corticotomy is useful in increasing tooth movement rate.^5^

Originally, corticotomy was composed of buccal and lingual flap reflections, followed by bone decortication. Later, some modifications to the cortical cuts were performed, of which the recent more attractive and lower-trauma form was osteoperforation. Osteoperforations are defined as surgical operations in which flaps are reflected and cortical bone perforations are performed, while the cancellous bone is reserved.^6^

Köle^7^(1959) first proposed the theory of “bony block movement”. Based on this theory, Köle successfully accelerated tooth movement by conducting a corticotomy around the moving tooth. Later, Wilcko et al.^8^ (2001) indicated that the fundamental principle of accelerated tooth movement assisted by corticotomy might not be the phenomenon of “bony block movement”; instead, it was the regional acceleratory phenomenon (RAP). Since then, RAP is supposed to be the rationale behind the accelerated effect of tooth movement after corticotomy.

RAP is a transient, sharp reconstruction and healing response for the local injured tissues. After a fracture, bone anabolic and catabolic activities around the injury site are accelerated. In detail, RAP can accelerate bone remodeling by recruiting osteoclasts and osteoblasts to the wound site.^9^ Later, alveolar bone density was decreased due to the localized increased bone remodeling.

Several researchers have demonstrated that corticotomy could shorten the orthodontic treatment period, but the results from clinical and animal studies indicated that the acceleration effects on the rate of tooth movement from corticotomy were inconsistent.^10,11^ In addition, considering the effects of different force magnitudes on the biological response of alveolar bone, the tooth movement results under the combined application of osteoperforation, and different traction force magnitudes have not been thoroughly studied.^12,13^ Furthermore, the suitable force under the application of osteoperforation needs to be reassessed to achieve faster tooth movement.

This study aimed to compare orthodontic tooth movement and histological responses with three different force magnitude designs under osteoperforation in rabbit models.

## Methodology

The animal model design was approved by the Animal Ethical and Welfare Committee of Sir Run Run Shaw Hospital, Zhejiang University School of Medicine (No. SRRSH2020818).

The experiments were based on the National Institutes of Health guide for the care and use of laboratory animals (NIH Publications No. 8023, revised 1978).

The experiment sample consisted of 48 male New Zealand Rabbits, weighing 2.2-2.8 kg (Xinjian, Xinchang, Zhejiang, China). The right mandibular first premolar was designed as the surgical side, and the left side was designed as the control side. The rabbits were randomly divided into three groups of 16 rabbits each, and three different interventions were made to the mandibular first premolar: the traction force magnitude was 50 g (Group A), the traction force magnitude was 100 g (Group B), and the traction force magnitude was 150 g (Group C).

### Surgical Procedure

A modified osteoperforation procedure was performed based on two previous studies.^14,15^ The rabbits were operated under general anesthetic by an intravenous ear injection of pentobarbital (Xitang, Shanghai, China; 3%, 1 mL/kg). A 10.0 mm intrasulcular incision was made, and a full-thickness flap on the buccal and lingual sites of the right first premolar region was folded. Three perforations in the mesial buccal alveolar bone and three perforations in the mesial lingual alveolar bone of the right mandibular first premolar were created with a 1.0-mm-diameter pilot drill bur at 250 rpm. The perforation penetrated the whole cortical layer and reached the medullary bone. The process was conducted under sufficient rinsing of 9% sodium chloride. The gingiva was subsequently sutured with absorbable thread.

**Figure 1:**
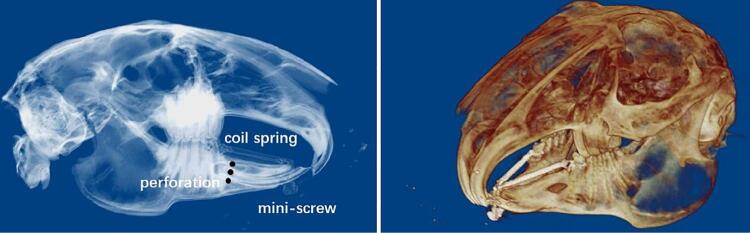
Osteoperforation-assisted tooth movement in rabbit models

A retentive horizontal groove around the crown of the mandibular first premolars was made using a slow-speed handpiece. One mini-screw (Protect, Hangzhou, Zhejiang, China) was inserted into bones between two mandibular central incisors using a manual driver. One nickel-titanium closed coil spring (Protect, Hangzhou, Zhejiang, China) was fixed to the right first premolar and the mini-screw with a 0.010-inch stainless steel ligature wire (Protect, Hangzhou, Zhejiang, China). Another coil spring was fixed to the first left premolar and the mini-screw (Figure 1). The coil spring would create 50 g/100 g/150 g continuous traction force; force was standardized with a dynamometer.

### Measurement of Tooth Movement Distance

**Figure 2:**
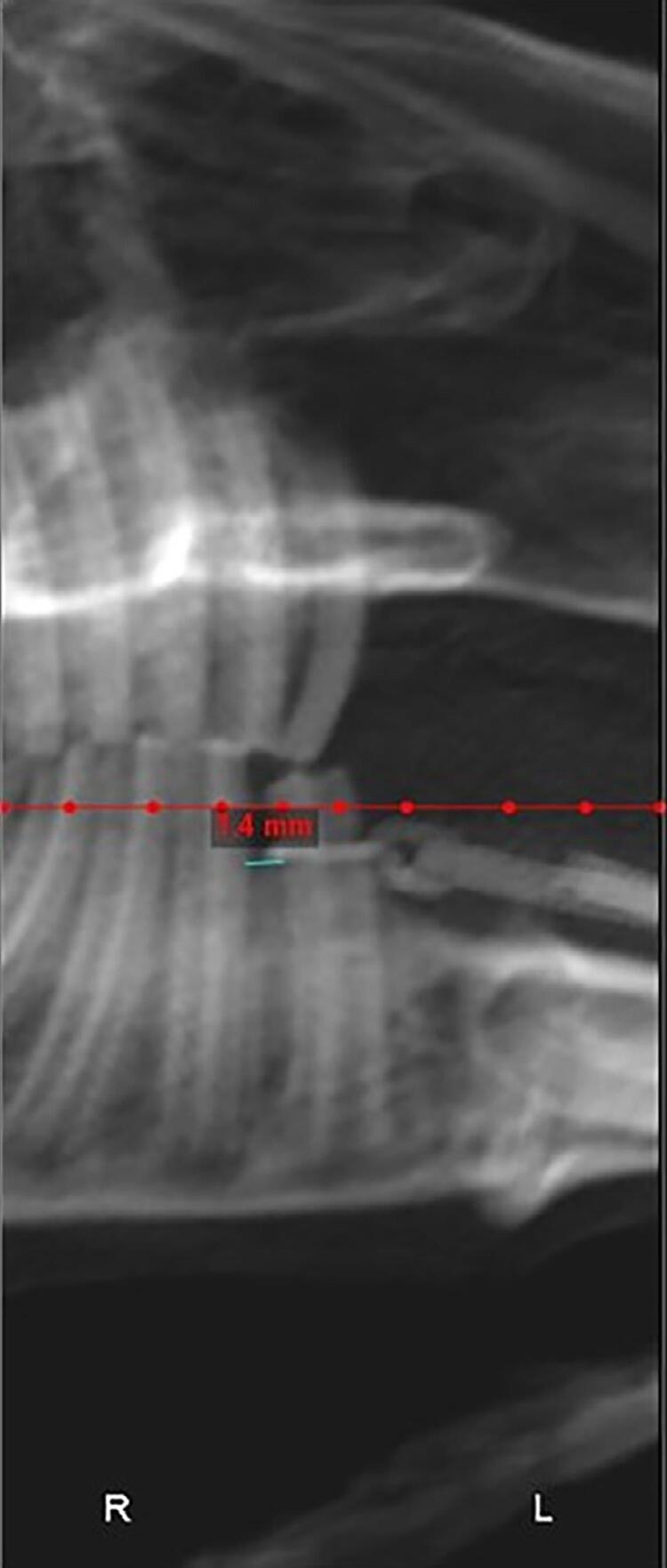
Computed tomography imaging for tooth movement distance measurement

The tooth movement distance was acquired from the three-dimensional CT data of the rabbits. The mandible of rabbits was scanned before surgery (T1) and after two weeks of spring traction (T2). CT data were uploaded into Dolphin software (Version 11.9, Dolphin Digital Imaging, Chatsworth, Calif, USA). The distance between the mesial alveolar crest of the second premolar and the distal alveolar crest of the first premolar on both sides was measured by a digital ruler at T1 and T2 (Figure 2). Tooth movement distance was calculated by subtracting the measured distance at T1 from the measured distance at T2. Three independent investigators conducted the measurement, and the average of the values acquired by three investigators was obtained for further analysis.

### Histological Staining

After two weeks, the rabbits were sacrificed by overdosing sodium pentobarbital. The specimens from the mandibular second premolars extending anteriorly to 10 mm mesial of the mandibular first premolars were obtained. The specimens were fixed in 10% formaldehyde, decalcified in 10% ethylenediaminetetraacetic acid (EDTA-2Na, pH 7.4), dehydrated with graded ethanol and embedded in paraffin. Then, the specimens were sectioned into 5-μm-thick sections horizontally parallel to the first premolar’ occlusal plane (SP 1600, Leica, Jena, Germany), and the intervals were 100 μm. The sections from the middle one-third of the premolar roots were selected and stained with hematoxylin and eosin (H&E) to examine overall morphology.

Sections were observed under a microscope (Eclipse 50i, Nikon, Tokyo, Japan), and images of the slices were captured under 40x and 100x magnification with digital camera software (NIS-Elements AR 3.1, Nikon, Tokyo, Japan).

### Immunohistochemical Staining

The sections from the middle one-third of the premolar roots in each specimen were stained with immunohistochemical staining to examine bone anabolic activity. Prepared sections were deparaffinized with xylene and hydrophilized with ethanol. The following primary antibodies were used: anti-PCNA (proliferating cell nuclear antigen, 1:200 concentration, Cell Signaling Technology, Boston, MA, USA), anti-OCN (osteocalcin, 1:100 concentration, Cell Signaling Technology, Boston, MA, USA), anti-VEGF (vascular endothelial growth factor, 1:100 concentration, Cell Signaling Technology, Boston, MA, USA), and anti-TGF-β1 (transforming growth factor beta 1, 1:100 concentration, Cell Signaling Technology, Boston, MA, USA). The sections were incubated with the primary antibodies at 48ºC overnight. Primary antibodies were diluted with sterile phosphate-buffered saline (PBS). Anti-rabbit IgG (Cell Signaling Technology, Boston, MA, USA) was used as the secondary antibody. Sections were incubated for 2 hours at room temperature with the secondary antibodies and then washed with PBS.

Image-Pro Plus 6.0 (Media Cybernetics, Rockville, Md, USA) was used to quantitatively measure the expression intensity of PCNA, OCN, VEGF, and TGF-β1. Ten-times magnified light microscopy images of the tension area (distal side of the first premolar) of alveolar bone were used to evaluate the labeling of PCNA, OCN, VEGF, and TGF-β1. The labeling was calculated as the mean optical density in each image. Three independent investigators conducted the measurement, and the mean values of the optical density obtained by three investigators were calculated for further data analysis.

### Statistical Analysis

All data are represented as the mean values ± standard deviations and were analyzed using SPSS 19.0 software (IBM Corp., Armonk, USA). The consistency of the three calculations of tooth movement distance and optical density were tested by the intraclass correlation (ICC). Exploratory chi-square statistical tests were conducted to examine normal distribution of tooth movement distance and mean optical density in the three groups.

An independent-samples T-test was performed to compare tooth movement distance and mean optical density between the surgical and control sides in each group. Additionally, an independent-samples T-test was conducted to compare tooth movement distance and mean optical density separately in the surgical side and the control side among the three groups. Statistical significance was tested at the levels of P<0.05, P<0.01 and P<0.001.

## Results

During the experiment, all springs remained intact. The mesial moving of the mandibular first premolars on both sides were observed in the three groups and all the premolars moved in the proper direction.

### Tooth Movement Distance and Rate

The ICC coefficients of tooth movement distance measurements were higher than 0.851, and the 95% confidence interval ranged from 0.656 to 0.993 among the measurements. The results of the exploratory chi-square statistical tests confirmed the hypothesis of normality of distribution for the tooth movement distance. The tooth movement distance on the surgical side was larger than the control side in all groups (P<0.01) (Table 1). We found no significant intergroup difference in tooth movement distance among the three groups (P>0.05) for the surgical side. For the control side, the tooth movement distance in Group A was significantly smaller than those in Group B (P<0.001) and Group C (P<0.001). However, we found no significant difference in tooth movement distance between Group B and Group C (P>0.05) for the control side.

**Table 1:** Distance of tooth movement after 2 weeks (mm)

	Control Side	Surgical Side	P
GA	0.52±0.08	1.02±0.17	0.000^‡^
GB	0.74±0.13	1.02±0.14	0.000^‡^
GC	0.73±0.14	1.03±0.27	0.001^†^
PAB	0.000^‡^	0.947*	
PAC	0.000^‡^	0.878*	
PBC	0.735*	0.834*	

Data are presented as mean±SD; PAB: the difference of tooth movement distance between Group A and Group B; PAC: the difference of tooth movement distance between Group A and Group C; PBC: the difference of tooth movement distance between Group B and Group C; P<0.05, P<0.01 and P<0.001=significant; *: P<0.05; ^†^: P<0.01; ^‡^: P<0.001.

The average movement rate of the premolars during the beginning two weeks was calculated to analyze the tooth movement in detail. In Group A, the tooth movement rate on the surgical side was 0.073 mm/day, and the rate on the control side was 0.037 mm/day. In Group B, the tooth movement rate on the surgical side was 0.073 mm/day, and the rate on the control side was 0.053 mm/day. In Group C, the tooth movement rate on the surgical side was 0.074 mm/day, and the rate on the control side was 0.052 mm/day.

### Histological Analysis

**Figure 3:**
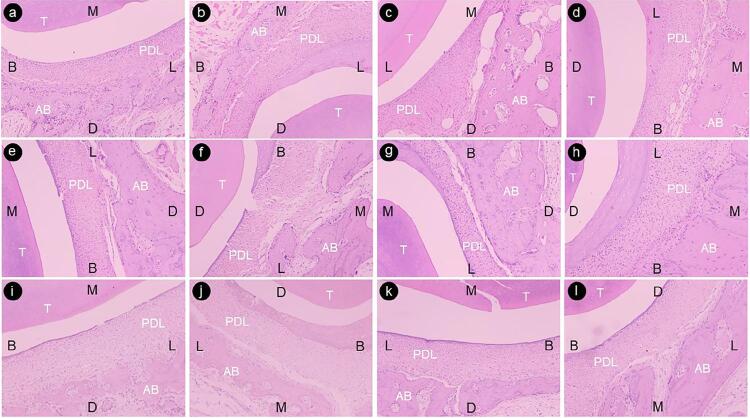
Microphotograph of the distal periodontal tissues of the first premolar with H&E staining (×40). a, the tension area, the control side, Group A; b, the compression area, the control side, Group A; c, the tension area, the surgical side, Group A; d, the compression area, the surgical side, Group A. e, the tension area, the control side, Group B; f, the compression area, the control side, Group B; g, the tension area, the surgical side, Group B; h, the compression area, the surgical side, Group B. i, the tension area, the control side, Group C; j, the compression area, the control side, Group C; k, the tension area, the surgical side, Group C; I, the compression area, the surgical side, Group C. T, tooth; PDL, periodontal ligament; AB, alveolar bone. B, buccal; D, distal; L, lingual; M, mesial.

We observed alveolar osteopenia on the compression area of the moving premolar in all groups. Visible periodontal cells and multinucleated osteoclast-like cells could be observed on the alveolar bone margin adjacent to the compressed periodontal ligament (PDL). We noted dilated blood vessels on the tension area of the moving premolar and newly formed woven bone with numerous osteocytes. (Figure 3).

### Immunohistochemical Analysis

The ICC coefficients of mean optical density measurements were higher than 0.561, and the 95% confidence interval ranged from 0.411 to 0.999 among the measurements. The results of the exploratory chi-square statistical tests confirmed the hypothesis of normality of distribution for the mean optical density. The data of immunohistochemical calculations in the three groups are presented in Table 2.

**Table 2:** Mean optical density of the immunohistochemical items on the tension area of the moving premolar

	PCNA			OCN			VEGF			TGF-β1		
	Control Side	Surgical Side	P	Control Side	Surgical Side	P	Control Side	Surgical Side	P	Control Side	Surgical Side	P
GA	0.065±0.002	0.076±0.002	0.000^‡^	0.141±0.004	0.221±0.004	0.000^‡^	0.009±0.007	0.012±0.005	0.000^‡^	0.155±0.002	0.230±0.002	0.000^‡^
GB	0.070±0.002	0.075±0.001	0.000^‡^	0.183±0.004	0.225±0.003	0.000^‡^	0.012±0.005	0.014±0.004	0.000^‡^	0.183±0.001	0.254±0.002	0.000^‡^
GC	0.070±0.001	0.076±0.001	0.000^‡^	0.184±0.003	0.225±0.002	0.000^‡^	0.012±0.005	0.014±0.003	0.000^‡^	0.183±0.002	0.254±0.002	0.000^‡^
PAB	0.000^‡^	0,47		0.000^‡^	0.006^†^		0.000^‡^	0.000^‡^		0.000^‡^	0.000^‡^	
PAC	0.000^‡^	0,704		0.000^‡^	0.002^†^		0.000^‡^	0.000^‡^		0.000^‡^	0.000^‡^	
PBC	0,542	0,585		0,313	0,713		0,206	0,114		0,549	0,945	

Data are presented as mean±SD; PAB: the difference of mean optical density between Group A and Group B; PAC: the difference of mean optical density between Group A and Group C; PBC: the difference of mean optical density between Group B and Group C; P<0.05, P<0.01 and P<0.001=significant; *: P<0.05; ^†^: P<0.01; ^‡^: P<0.001.

**Figure 4:**
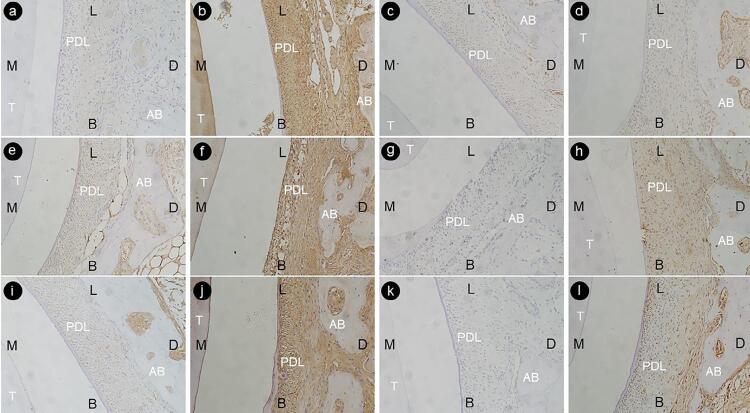
Immunohistochemical staining of PCNA, OCN, VEGF and TGF-β1 on the tension area of the moving premolar of the control side (×200). a, PCNA, Group A; b, OCN, Group A; c, OCN, Group A; d, VEGF, Group A. e, PCNA, Group B; f, OCN, Group B; g, OCN, Group B; h, VEGF, Group B. i, PCNA, Group C; j, OCN, Group C; k, OCN, Group C; l, VEGF, Group C.

We found PCNA, OCN, VEGF, and TGF-β1 signals on the tension area of the moving premolar in the alveolar bone in all groups (Figure 4). The expression levels of PCNA, OCN, VEGF, and TGF-β1 on the surgical side were more expressive than the control side in all groups (P<0.001).

## Discussion

We designed and modified the osteoperforation procedure from previous studies. Many studies designed diameter 0.25 mm or 0.5 mm to osteoperforations in Wistar rat models.12,16-20 As for New Zealand white rabbit models, Kim et al.15 (2019) designed diameter 1.0 mm and 1.4 mm to osteoperforations. Based on these, we selected diameter 1.0 mm to osteoperforations in rabbit models. In one study, Kurohama et al.14 (2017) reported that the corticotomy was performed to the rats’ maxilla using a round bur at a low speed of 100 rpm. Considering the density of the mandibular cortical bone, we supposed that the rpm should be higher for rabbit models than for rat models. Combined with the factor of injury to the bone during the drilling procedure, we finally selected 250 rpm to osteoperforations in rabbit models.

The mandibular first premolar movement model applied in our study was improved from the model used formerly. As rabbits have small incisors with poor retention, a mini-screw inserted into the bone between two mandibular incisors was used as an anchor unit. We used a nickel-titanium coil spring-assisted by the mini-screw to pull the mandibular first premolar and move it mesially.

Several studies have indicated that osteoperforation could accelerate orthodontic tooth movement.^15,21^ In our experiment, the amounts of tooth movement on the surgical side were more extensive than those on the control side by 96.2%, 37.8%, and 41.1%, respectively, in Groups A, B, and C. Consistent with our findings, Chen, et al.^22^ (2016) stated an evident difference in tooth movement distance between the corticotomy group (0.89 mm) and the control group (0.80 mm) after 14 days of traction in rabbit models. However, in disagreement with our findings, Alkebsi, et al.^23^ (2018) claimed no noticeable difference in tooth movement distance between the osteoperforation and the control groups. Differences between these studies might be due to differences in the study design, such osteoperforation itself, bone removal associated with osteoperforation, animal model, and magnitude of traction force.

The tooth movement rate can be regulated by the degrees of bone turnover, bone density and PDL hyalinization in response to the traction force.^24^ Regarding bone turnover, the balance between bone regeneration and bone resorption during tooth movement will affect the rate of bone remodeling and subsequently influence the rate of tooth movement. Our results were consistent with previous studies^25,26^ showing greater bone apposition and increased localized bone turnover subjected to corticotomy and resulted in the acceleration of tooth movement.

We discovered that osteoperforation-assisted tooth movement distance under different force magnitudes was almost similar. We then inferred that the injury from traction force was much lower compared with cortical bone perforation, and the increase of force magnitude in the movement of osteoperforation-assisted teeth would not increase alveolar bone resorption.^17^ This discovery was similar to Zuppardo et al.^26^ (2020), who reported that adding decortication to corticotomy would not improve its efficiency of accelerating tooth movement. Therefore, a low force magnitude of 50 g was enough to benefit fast osteoperforation-assisted tooth movement.

The immunohistochemical staining analysis can help us better understand the change in osteoblast activities on the tension area of the moving tooth. PCNA is a cell cycle-associated nuclear protein that is maximally elevated in proliferating cells, and it is reportedly useful for assessing the proliferation activity of osteoblasts.^27^ OCN is a mature marker of osteoblasts and present at high levels in a mineralized bone matrix during the late stage of bone formation.^28^ In our experiment, after two weeks of osteoperforation, the mean optical densities of PCNA-positive osteoblasts and OCN-positive osteoblasts on the surgical side were significantly higher than the control side in all groups. However, there was no difference in the expression of PCNA under osteoperforation among the three groups. These discoveries indicated that osteoblasts around the moving tooth were active at the early stage under osteoperforation, and a lower force of 50 g was enough to activate the osteoblasts.

Based on the osteoinductive and vasculogenic properties of growth factors, VEGF and TGF-β1 play important roles in the differentiation and mineralization of osteoblasts.^29^ It has been demonstrated that osteoperforation could increase the expression of VEGF and TGF-β1 around the moving tooth and promote alveolar bone remodeling.^30^ In our study, increased expression levels of VEGF and TGF-β1 were observed around the moving tooth under osteoperforation compared with the control side.

This study has some limitations. First, longer observation times are lacking. Further research with a comparative approach as reported in this study and longer observation periods are necessary to determine whether the difference in tooth movement distance and biological response will continue to evolve. Moreover, the lack of micro-CT examination caused debates about the precise response of bone remodeling. Further studies applying micro-CT examination might be useful to provide more information.

## Conclusions

1. The application of osteoperforation could accelerate the orthodontic tooth movement rate in rabbits.
2. An application of a light force of 50 g was suitable to achieve fast osteoperforation-assisted tooth movement in rabbits.
